# Rapidly progressive destructive arthrosis (RPDA) of the glenohumeral joint: a radiologic classification and mid-term outcomes following shoulder arthroplasty

**DOI:** 10.1186/s12891-025-09283-7

**Published:** 2025-12-24

**Authors:** Bo-Seoung Kim, Byung-Uk Song, Sang-Min Lee, Seong-Hun Kim, Jin-Kyu Kang, Jong-Hun Ji

**Affiliations:** 1 Department of Orthopaedic Surgery, Daejeon St. Mary’s Hospital, College of Medicine, The Catholic University of Korea, 64, Daeheung-ro, Jung-gu, Daejeon, 34943 Republic of Korea; 2 Department of Orthopaedic Surgery, Seoul Daejeong Hospital, Chun-an, Korea; 3 Department of Orthopaedic Surgery, National Health Insurance Service Ilsan Hospital, Goyang-si, Korea

**Keywords:** Rapidly progressive destructive arthrosis, Shoulder arthroplasty, Humeral head collapse, Reverse total shoulder arthroplasty, Glenohumeral joint

## Abstract

**Purpose:**

Rapidly progressive destructive arthrosis (RPDA) of the glenohumeral joint is a rare but distinct clinical entity characterized by rapid humeral head collapse of unclear etiology. This study aimed to characterize the clinical, radiologic, and intraoperative features of RPDA, to propose a three-stage radiologic classification, and to evaluate short-term outcomes following shoulder arthroplasty (reverse or total) according to disease stage.

**Methods:**

From March 2015 to December 2023, 23 patients were diagnosed with RPDA of the glenohumeral joint based on clinical and radiologic findings. After excluding those with alternative etiologies, 19 patients who underwent shoulder arthroplasty (17 reverse total shoulder arthroplasties and 2 anatomic total shoulder arthroplasties) were included in tha analysis. Patients were classified into three radiologic stages (early, mid, and late RPDA) and evaluated at a minimum follow-up of two years.

**Results:**

All 19 patients were female, with a mean age of 73.8 ± 7.9 years. The mean duration of symptoms before humeral head collapse was 14.7 ± 18.4 months (range, 3–60 months). Patients were classified into three distinct radiologic stages based on imaging findings. Preoperatively, 47% (9/19) had a history of trauma or prior rotator cuff surgery. At the final follow-up, all patients showed significant improvement in clinical outcomes. Postoperative ASES and SST scores, as well as preoperative symptom duration, differed significantly among the three stages (*p* < 0.05). No significant differences were observed in postoperative range of motion or complication rates. Two complications occurred: one acromial fracture and one periprosthetic humeral shaft fracture.

**Conclusion:**

RPDA of the shoulder often progresses rapidly and may be overlooked in its early stages. Radiologic staging with MRI and plain radiography facilitates timely diagnosis and appropriate surgical planning. Shoulder arthroplasty—either reverse or anatomic TSA—resulted in favorable clinical and radiological outcomes, particularly when performed before advanced humeral head collapse or glenoid involvement. Early recognition and timely intervention are therefore essential to optimize patient outcomes.

**Level of evidence:**

Level 4, case series study.

## Introduction

Rapidly progressive destructive arthrosis (RPDA) of the glenohumeral joint is a distinct yet poorly understood condition, and its underlying pathophysiology remains uncertain.Although RPDA is rare, its incidence appears to be increasing with the aging population. RPDA refers to a group of disorders characterized by the rapid destruction of the shoulder joint. The terms rapidly destructive arthropathy (RDA), very rapidly progressive shoulder arthropathy (VRPSA), rapid progressive osteonecrosis (RPON), and rapidly progressive osteoarthritis (RPOA) are sometimes used interchangeably, although their definitions may vary slightly depending on clinical context. Various underlying diseases, such as rheumatoid arthritis (RA), septic arthritis, osteonecrosis (ON), and crystalline arthropathy, may lead to the development of RPDA. Smith and Adams [1] were the first to describe the rapidly destructive arthrosis of the shoulder. Characteristic radiologic features can be identified on the radiologic findings such as X-ray, CT or magnetic resonance imaging (MRI). However, the radiologic manifestations of RPDA are often misinterpreted as arthropathies, infection, osteonecrosis or insufficiency fracture.

The joint most frequently affected by RPDA is the hip joint [2–5]. The typical radiological findings included flattening of the femoral head, articular cartilage loss, subchondral bone destruction and joint effusion. Previous reports have suggested that surgeons should consider the presence of RPDA when patients show rapid femoral head destruction [6]. These studies on RPDA in the hip joint showed the distinct clinical features and successful treatments which lead to the treatment of RPDA. In the shoulder joint, RPDA is characterized by rapid progressive collapse of the humeral head without evidence of any underlying pathology. The involvement of hip and knee is more common than the shoulder joint [4, 7, 8]. Although the hip and knee are more commonly involved than the shoulder, shoulder involvement remains relatively rare. There are various theories for its pathophysiology, but among them, crystal-induced arthritis is considered to be a possible source for shoulder disease. Hydroxyapatite crystals have been detected in the synovium of shoulders affected by rapidly progressive arthritis (Milwaukee shoulder) as first described by McCarty et al. [9] in 1981 and are considered to be a trigger for inflammation leading to joint destruction [10]. RPDA most often occurs in elderly women, but sometimes, also seen in patients after sustaining trauma. The diagnosis of RPDA mainly depends on clinical and radiologic features. Both radiologists and clinicians should be aware of this entity to avoid unnecessary diagnostic work-up and to ensure timely and appropriate treatment.

In patients with RPDA, advanced age and poor-quality rotator cuff tendons were common findings. In many cases, the collapsed humeral head was accompanied by absence of rotator cuff tissue. Among several treatment options of these destructive arthrosis, shoulder arthroplasty represents one of the most effective approaches. Even in the presence of a large proximal humeral bone defect, shoulder arthroplasty, especially reverse total shoulder arthroplasty (rTSA) could be a good treatment solution. However, few studies have reported on about the treatment of shoulder arthroplasty of these specific disease entity.

Therefore, the purpose of this study was to evaluate clinical course, radiologic and intraoperative findings of RPDA of the glenohumeral joint. The hypothesis was that shoulder arthroplasty would yield favorable clinical and radiologic outcomes at minimum 2-year follow-up.

## Materials and methods

From March 2015 to March 2023, 23 patients were diagnosed with rapidly progressive destructive arthrosis (RPDA) of the shoulder at our institution. Clinical diagnosis of RPDA was based on severe shoulder pain with rapid functional deterioration, often progressing to pseudoparalysis within months, in combination with radiographic evidence of rapid humeral head collapse. The diagnosis of RPDA was made according to the definition proposed by Lequesne [11], in which rapid progression is defined as a loss of more than 2 mm of joint space per year or a 50% reduction of joint space within one year, in the absence of another identifiable cause of destructive arthropathy [11]. Inclusion criteria were radiologic evidence of RPDA on plain radiography, CT, or MRI, and all patients provided informed consent prior to participation in the study. The diagnosis of RPDA was otherwise made by exclusion. Septic arthritis was ruled out by normal inflammatory markers, and osteonecrosis was excluded not only by the absence of common risk factors such as steroid use or alcohol abuse but also by consideration of other possible causes, including chemotherapy, lupus erythematosus, and sickle cell disease, together with the absence of characteristic imaging findings on MRI or CT and relevant clinical features. Neuropathic arthropathy was excluded based on detailed clinical history and neurologic examination, rheumatoid arthritis was excluded by serologic testing and clinical assessment, and crystal-induced arthropathy was excluded by the absence of typical histologic findings. Nearly half of the patients indeed had a history of prior trauma or arthroscopic rotator cuff repair; however, after careful review, none of these cases demonstrated radiologic or clinical features consistent with post-traumatic osteoarthritis, osteonecrosis, or chondrolysis following arthroscopy. To clarify this differential diagnosis, we carefully reviewed imaging, clinical, and histologic features.

Post-traumatic osteoarthritis typically progresses gradually over years with osteophyte formation and subchondral sclerosis, whereas RPDA demonstrates rapid humeral head collapse within months and focal-to-diffuse bone marrow edema or subchondral fracture bands on MRI/CT. Osteonecrosis was excluded by the absence of major risk factors such as chronic steroid use, alcoholism, or autoimmune disease, and no patient showed the serpiginous necrotic line typical of avascular necrosis on T1 MRI. Post-arthroscopy chondrolysis was ruled out because all patients lacked exposure to intra-articular anesthetic pumps or thermal injury, and imaging did not show isolated cartilage loss with preserved subchondral bone. Instead, our cases revealed subchondral insufficiency fractures and marrow edema leading to rapid humeral head collapse, with histology showing reactive synovitis and fracture-healing callus rather than toxic cartilage dissolution. Of the 23 patients identified, 19 underwent shoulder arthroplasty and were included in the study. Four patients were managed conservatively because of medical comorbidities or refusal of surgery. Among the 19 surgical patients, 17 underwent reverse total shoulder arthroplasty (RTSA), and 2 underwent anatomic total shoulder arthroplasty (TSA). All patients were followed for a minimum of two years. Demographic data are summarized in Table 1. Clinically, patients typically presented with pseudoparalysis or painful restriction of shoulder motion. Early diagnosis was often challenging because radiographic findings in the initial stages were subtle and nonspecific. We retrospectively classified patients into three groups based on radiologic findings from plain radiography and magnetic resonance imaging (MRI):

- Group 1 [early (chondrolysis) stage] : Four patients showed early head collapse. Imaging revealed focal subchondral bone edema on MRI and small subchondral fractures on X-ray. All patients had partial or full-thickness rotator cuff tears with poor tendon quality. Arthroscopy revealed necrotic cartilage, embedded fibrotic tissue, and synovial hypertrophy
- Group 2 [mid (subchondral destruction) stage] : Nine patients showed progressive humeral head collapse involving less than 50% of the articular surface of the humeral head. MRI revealed extensive bone marrow edema throughout the humeral head, which gradually subsided over time. All patients had full-thickness rotator cuff tears with poor tendon quality.
- Group 3 [late (subchondral destruction) stage]: Six patients exhibited humeral head collapse extending to or beyond the anatomic neck. All had massive, complete rotator cuff deficiency. RTSA with a long stem was preferred in these cases because of compromised metaphyseal bone stock.

**Table 1:** Patient demographics of rapidly progressive destructive arthropathy (RPDA)

	Patients
Number	19 patients
Sex	Female (all)
Age	72.6 ± 8.4 (55–87)
BMI	25.2 ± 4.4 (19.2–32.9)
Operated hand	Right (all)
Trauma History	9 cases including 3 arhroscopic rotator cuff repair
Symptom Duration (week)	19.1 ± 29.5 (3.0–100.0.0.0)
Laboratory findings (ESR/CRP)	9.06 ± 7.05 (0.08–20.08)/0.17 ± 0.14 (0.03–0.5)
Full thickness Rotator cuff tears	14 patients
Secondary glenoid bone involvement	7 patients

We collected data retrospectively from medical records, including symptom duration, trauma history, imaging features, and clinical outcomes. At final follow-up, functional outcomes were assessed using ASES, UCLA, and SST scores, as well as shoulder range of motion (ROM). Radiologic evaluation included assessment of component loosening, notching, and complications. Laboratory studies (ESR, CRP, rheumatoid factor) were performed to exclude infection, autoimmune disease, or neuropathic arthropathy. SPSS version 20.0 (IBM Corp., Armonk, NY, USA) was used for statistical analysis. Pre- and postoperative clinical scores and ROM were compared using the paired t-test. For group comparisons across the three radiologic stages, one-way ANOVA or the Kruskal-Wallis test was applied depending on normality assumptions, which were assessed using the Shapiro-Wilk test. Categorical variables were analyzed using the chi-square test. A *p*-value less than 0.05 was considered statistically significant.

## Results

Nineteen patients diagnosed with RPDA who underwent shoulder arthroplasty were included in the final analysis. All patients were female, with a mean age of 73.8 ± 7.9 years (range, 55–87). In every case, the dominant (right) shoulder was affected. The mean duration from symptom onset to radiographically confirmed humeral head collapse was 14.7 ± 18.4 months (range, 3–60 months). Half of the patients had a history of corticosteroid injection, and 47% (9/19) had prior trauma or arthroscopic rotator cuff repair. Among the 19 patients, 17 underwent RTSA and 2 underwent TSA. Despite the presence of an intact rotator cuff in some cases, poor tendon quality and fatty degeneration led to selection of RTSA over TSA in most patients. Eleven patients received a conventional (long-stem) humeral component, while eight received a short-stem implant. In cases with severe metaphyseal bone loss or subchondral insufficiency fractures (particularly in late-stage RPDA), long-stem implants were preferred for enhanced fixation. At final follow-up, all patients demonstrated significant improvement in clinical outcomes (Table 2).

**Table 2:** Comparison of preoperative and postoperative clinical outcomes in rapidly progressive destructive arthropathy (RPDA)

	Preoperative	Postoperative	*P*-value
Clinical score			
VAS	3.7 ± 0.8 (3.0–5.0)	0.8 ± 0.7 (0.0–2.0)	0.000
ASES score	26.5 ± 17.0 (10.0–65.0)	75.1 ± 18.5 (30.0–100.0.0.0)	0.000
UCLA score	10.5 ± 6.1 (4.0–24.0)	26.8 ± 6.3 (9.0–33.0)	0.000
SST score	2.0 ± 2.3 (0.0–8.0)	vs. 8.1 ± 3.1 (0.0–12.0)	0.000
Range of motion			
Forward flexion	98.9 ± 33.3 (60.0–140.0.0.0)	137.8 ± 10.9 (130.0–160.0.0.0)	0.003
Abduction	91.1 ± 41.1 (20.0–140.0.0.0)	138.9 ± 12.7 (130.0–160.0.0.0)	0.003
External rotation	12.2 ± 12.0 (0.0–30.0)	20.0 ± 10.0 (10.0–30.0)	0.211
Internal rotation	Lumbar vertebrae 5 (Buttock- Lumbar vertebrae 1)	Lumbar vertebrae 2(Lumbar 5-Thoracic vertebrae 12)	0.005

### Radiologic outcomes

Radiologic findings were carefully reviewed for staging of the lesions. Plain radiographs revealed humeral head collapse and flattening with osteolytic changes, which were apparent only in advanced cases. Based on combined imaging findings from plain radiographs, computed tomography (CT), and magnetic resonance imaging (MRI), patients were classified into three radiologic stages.

**Fig. 1:**
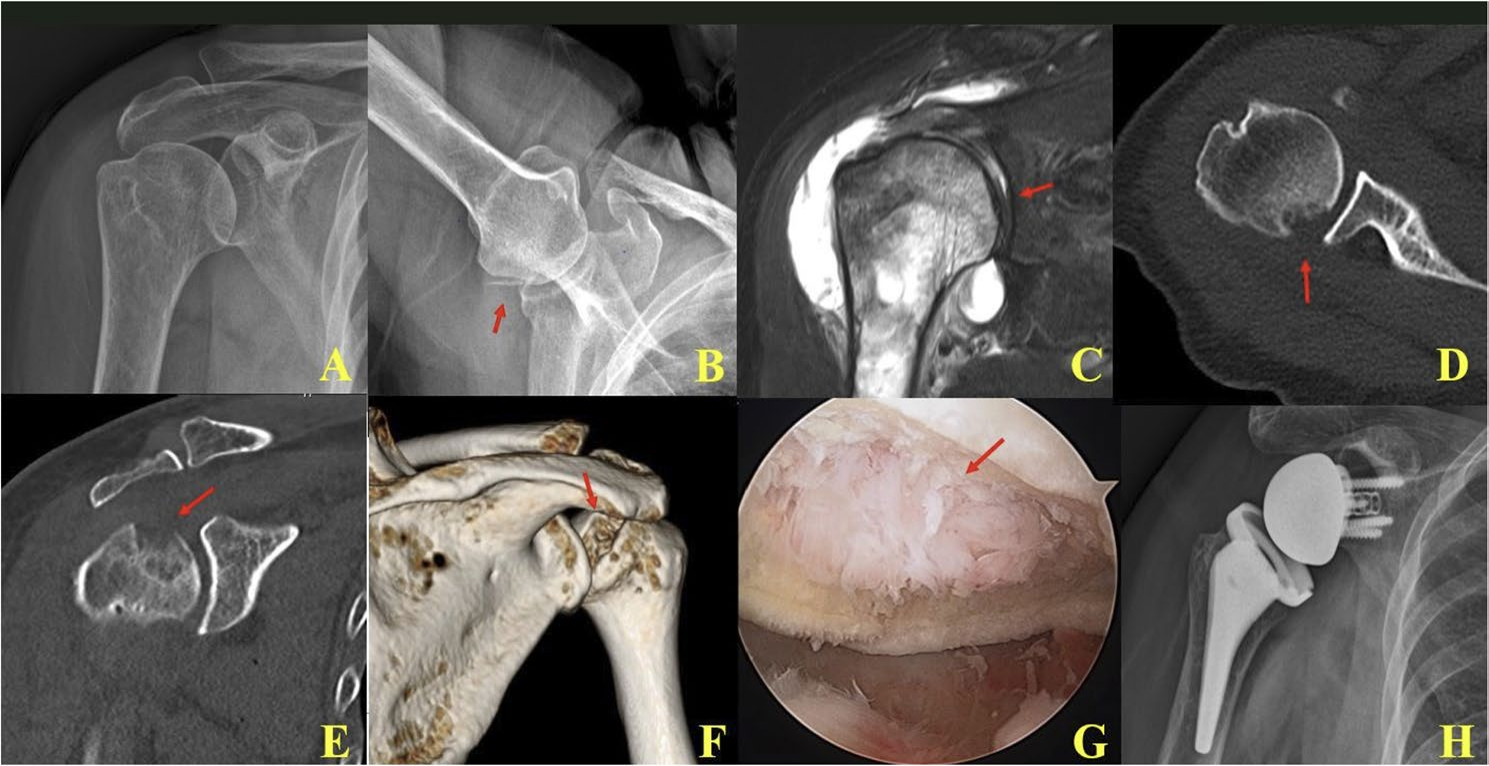
In the early prodromal phase of RPDA: **A**, **B** Initial plain radiographs demonstrated no abnormalities; however, follow-up imaging at six months revealed a subchondral fracture in the posterolateral aspect of the humeral head. **C** Initial MRI showed partial bone marrow edema of the humeral head, rather than diffuse involvement. **D**, **E** Follow-up CT scans at six months clearly demonstrated a subchondral fracture. (**F**) 3D CT revealed posterolateral collapse of the humeral head. **G** Arthroscopic evaluation showed complete loss of articular cartilage, embedded fibrotic tissue, and synovial hypertrophy. **H** Due to persistent shoulder pain, reverse total shoulder arthroplasty was performed one month later

**Fig. 2:**
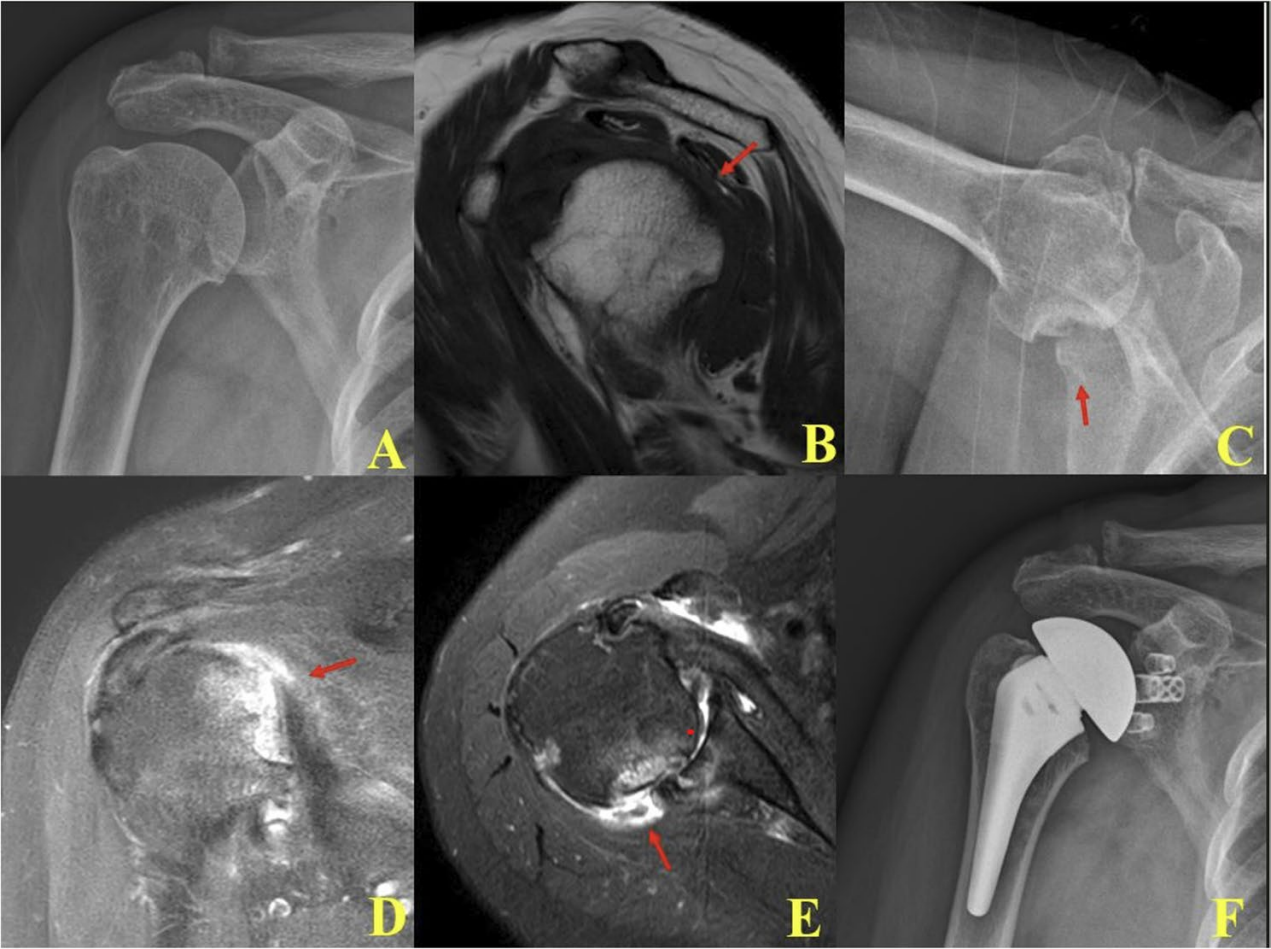
In the early prodromal phase of RPDA: **A** Initial plain radiograph showed no specific abnormalities of the humeral head. **B** Initial MRI revealed focal bone marrow edema, rather than diffuse involvement of the entire humeral head. **C** At six-month follow-up, plain radiograph demonstrated a newly developed focal subchondral fracture of the humeral head. **D**, **E** Follow-up MRI at six months confirmed the subchondral fracture without evidence of glenoid defects or rotator cuff tears. **F** Total shoulder arthroplasty was subsequently performed

In the prodromal (early) stage (Stage 1), MRI demonstrated focal bone marrow edema of the humeral head with an intact rotator cuff tendon. Some cases showed partial- or full-thickness but reparable tendon tears (Figs. 1 and 2).

**Fig. 3:**
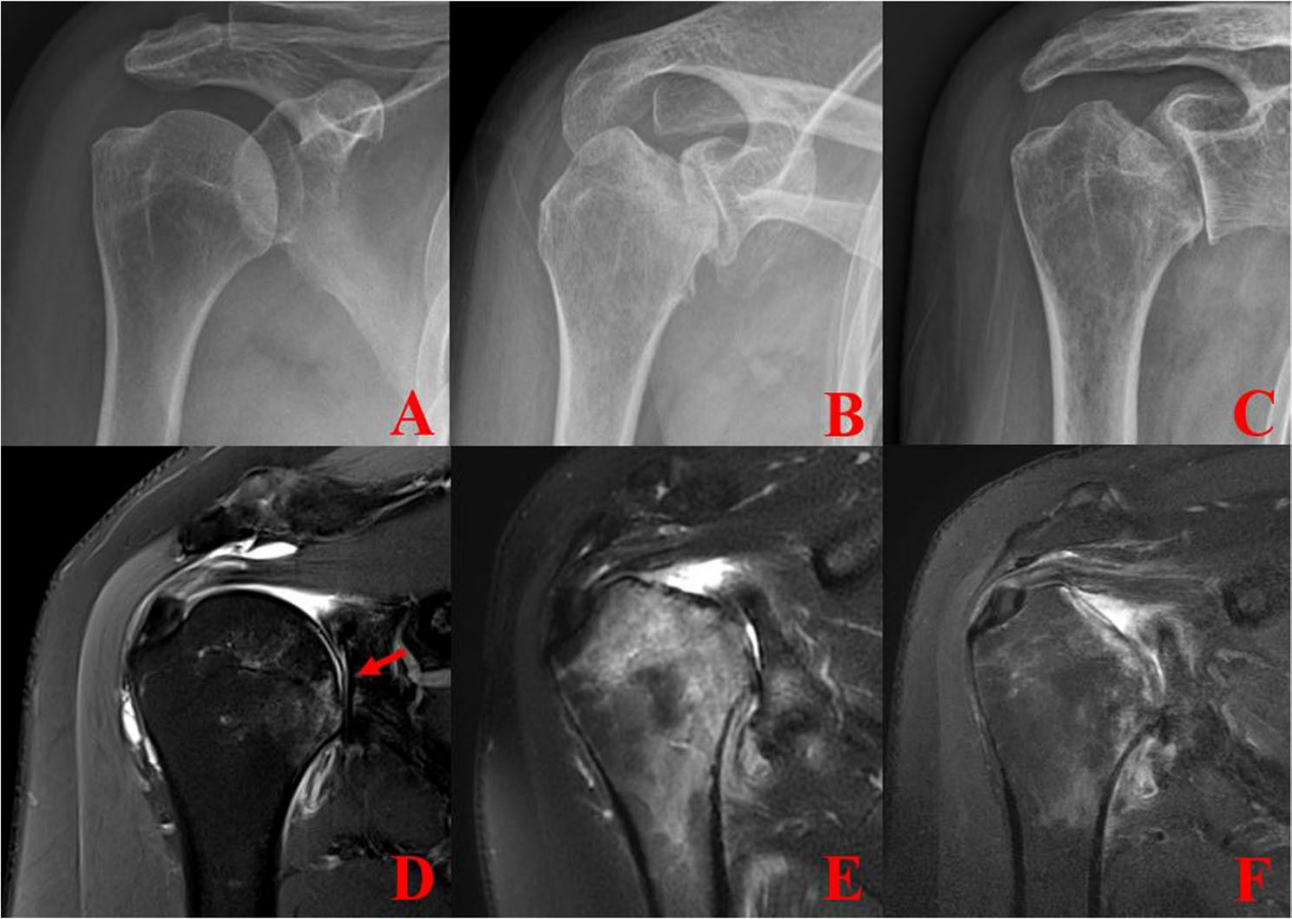
Mid-chondrolysis stage of rapidly progressive destructive arthropathy (RPDA): **A**, **D** Initial anteroposterior radiographs of the right shoulder taken at the first visit showed no apparent abnormalities of the humeral head. However, MRI revealed focal bone marrow edema in the humeral head (red arrow). **B**, **E** At the second visit, four months later, radiographs demonstrated progressive destruction of the humeral head, and MRI showed partial collapse with extensive bone marrow edema involving the entire humeral head. **C**, **F** Three months later, further progressive destruction was noted; however, the collapse did not exceed 50% of the humeral head. Follow-up MRI showed gradual resolution of the bone marrow edema over time

In the mid (subchondral destruction) stage (Stage 2), bone marrow edema had progressed to involve nearly the entire humeral head. Plain radiographs revealed subchondral collapse involving less than 50% of the articular surface. Interestingly, follow-up MRI showed that bone marrow edema gradually subsided over time, yet the degree of head collapse remained stable without further progression (Fig. 3).

**Fig. 4:**
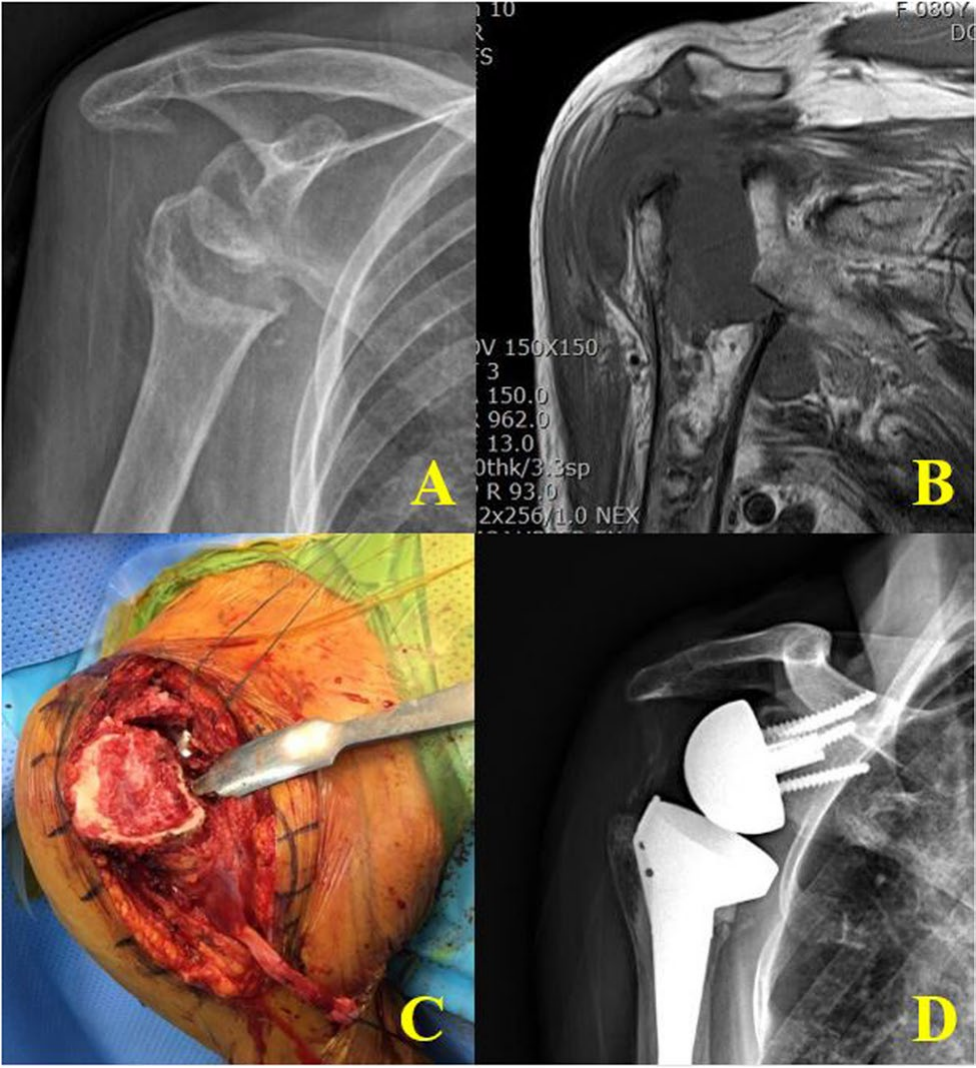
Representative findings in the late stage of a three-stage classification system for RPDA: **A **Anteroposterior radiograph of the right shoulder showing humeral head collapse, flattening, and osteolytic changes with severe osteopenia. **B** T1-weighted coronal MRI demonstrating an irreparable rotator cuff tear and significant joint effusion around the humeral head, with relative preservation of the glenoid. **C** Intraoperative image revealing massive bone loss with destruction of the humeral head extending to the humeral neck. **D** Postoperative radiograph showing reverse total shoulder arthroplasty

In the late (advanced destruction) stage (Stage 3), collapse of the humeral head exceeded 50% of the articular surface and was accompanied by proximal humeral metaphyseal bone loss. Radiographs and MRI demonstrated severe destructive arthrosis of the humeral head with multiple calcifications around the shoulder joint (Fig. 2). Prominent, progressive collapse of the humeral head was evident, indicating ongoing structural deterioration independent of marrow edema resolution. Among these advanced cases, three patients exhibited secondary glenoid defects or arthrosis due to humeral head destruction (Fig. 4).

### Clinical and histologic findings

Based on the combined radiologic findings from X-ray, CT, and MRI, patients were categorized into three stages according to the extent of humeral head destruction.

In the early stage (Stage 1), glenoid erosion and rotator cuff deficiency were not evident (Figs. 2 and 3). Radiologic and histologic findings demonstrated preserved humeral head architecture with small subchondral fractures visible on radiographs taken shortly after symptom onset. MRI confirmed focal bone marrow edema and early subchondral changes suggestive of microfracture. Arthroscopic observation revealed loss of articular cartilage, embedded fibrotic tissue, and synovial hypertrophy (Fig. 2). Total shoulder arthroplasty (TSA) was considered appropriate for this stage.

In the mid stage (Stage 2) (Fig. 4), MRI showed diffuse bone marrow edema throughout the humeral head that gradually decreased over time. Subchondral depression and partial collapse involving less than 50% of the articular surface were noted, but further progression was not observed. Histologic findings were consistent with the reparative phase of RPDA.

**Fig. 5:**
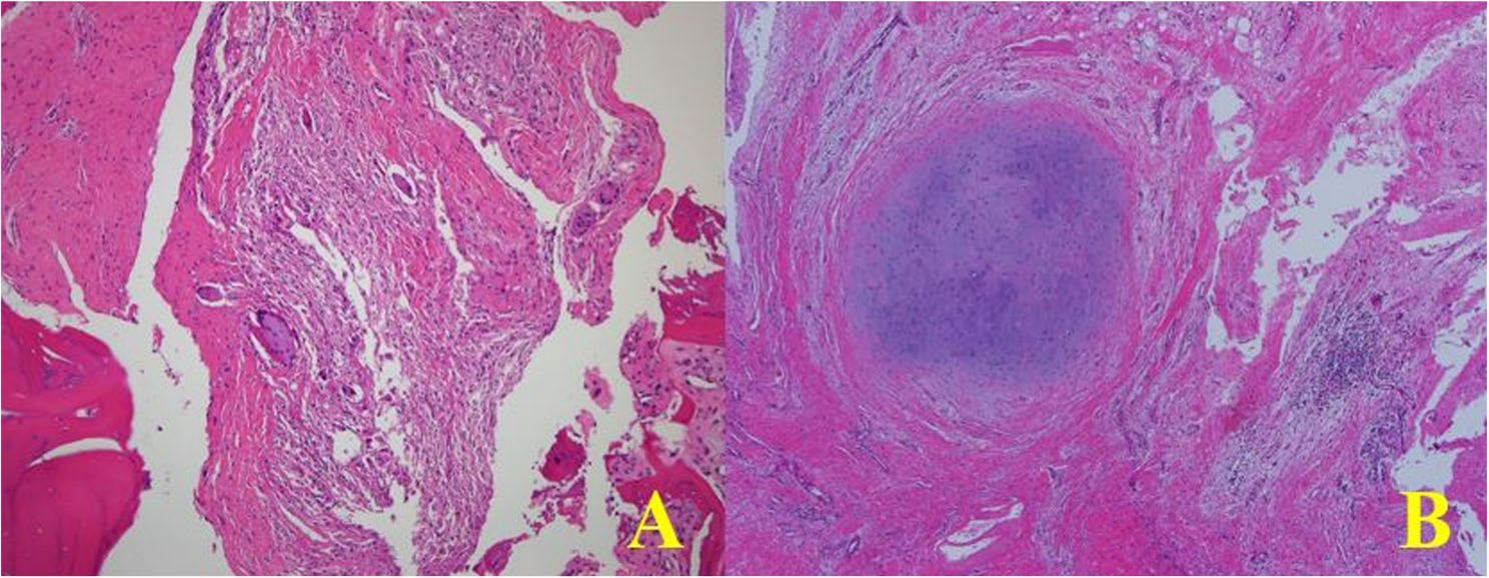
Histologic findings of rapidly progressive destructive arthropathy (RPDA): **A** Histopathological examination shows chronic inflammation with numerous macrophages, several foreign body-type multinucleated giant cells, and fibrosis (H&E stain, ×100). **B** Adjacent soft tissue demonstrates chondroid metaplasia and chronic inflammatory changes (H&E stain, ×400)

**Fig. 6:**
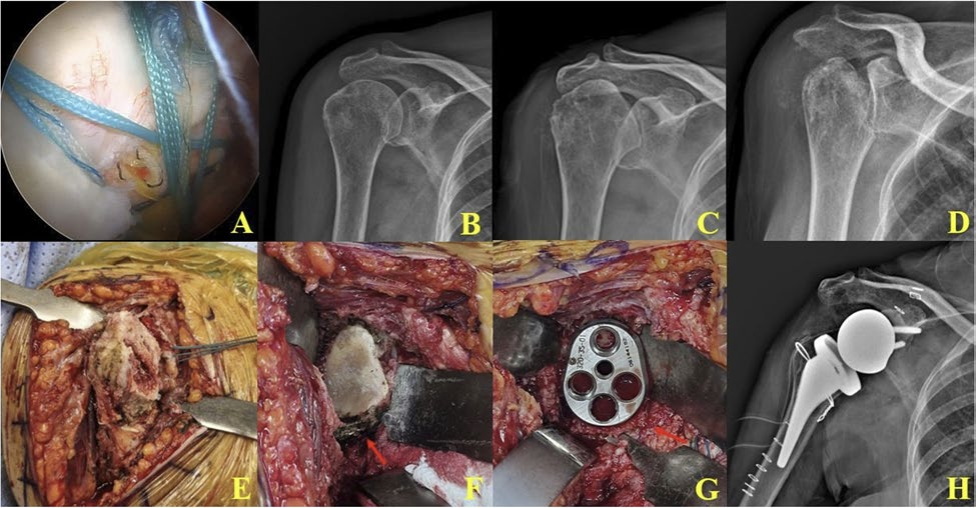
Sequential radiologic, arthroscopic, and intraoperative findings in a patient with rapidly progressive destructive arthropathy (RPDA) undergoing reverse total shoulder arthroplasty (RTSA). **A**, **B** Arthroscopic view six months prior shows a suture bridge rotator cuff repair, and plain radiographs demonstrate a normal glenohumeral joint without humeral head collapse. **C**, **D** Serial plain radiographs over a four-week period show progressive humeral head collapse and subchondral bone destruction. **E** Intraoperative photograph reveals extensive subchondral bone loss with severe humeral head destruction. **F** A secondary glenoid bone defect is observed intraoperatively (red arrow). **G** Glenoid baseplate fixation is performed with bone grafting (arrow) during the RTSA procedure. **H **Postoperative radiograph shows a well-positioned reverse total shoulder prosthesis

In the late (advanced) stage (Stage 3) (Figs. 5 and 6), collapse extended to the entire humeral head and metaphyseal region, accompanied by extensive subchondral bone loss and secondary glenoid erosion. In these cases, reverse total shoulder arthroplasty (RTSA) with a long stem was preferred because metaphyseal deficiency could not provide sufficient fixation for a short stem. Glenoid baseplate fixation often required bone grafting (Fig. 5).

Based on both radiologic and intraoperative findings, rotator cuff status varied with disease severity. In early-stage RPDA, most shoulders had intact or partial-thickness rotator cuff tendons, whereas advanced cases demonstrated extensive full-thickness or multi-tendon tears, reflecting secondary degeneration associated with humeral head collapse and superior migration.

Among the three radiologic stages, significant differences in clinical outcomes were observed. Postoperative SST scores and preoperative symptom duration differed significantly among the groups. Patients in Stage 3 showed lower postoperative functional scores (*p* = 0.025) and longer preoperative symptom duration compared with those in the early stage (*p* = 0.049). However, no significant differences were found in postoperative range of motion or complication rates among the three stages (Table 3).

**Table 3:** Comparison of clinical outcomes among the three radiologic stages of RPDA

	Stage 1	Stage 2	Stage 3	*p*-value
Age	70.83 ± 4.49	75.00 ± 6.52	71.40 ± 12.90	0.836
Sex	female	female	female	n.s
Symptom duration	5.00 ± 4.00	22.20 ± 43.51	39.20 ± 28.93	0.049
BMI	25.6 ± 4.21	26.06 ± 4.77	22.73 ± 4.76	0.739
Trauma History	57.0%	50.0%	33.0%	0.684
Complication	1 acromion stress fracture	0	1 periprosthetic humeral fracture	
Pre-operative				
VAS	3.86 ± 0.9	3.45 ± 0.69	3.83 ± 0.75	0.335
ASES	30.04 ± 23.29	23.15 ± 13.05	18.64 ± 9.97	0.527
UCLA	11.5 ± 8.04	9.7 ± 4.45	8.6 ± 3.71	0.630
SST	2.33 ± 2.94	1.7 ± 1.25	1.8 ± 1.48	0.634
Post-operative				
VAS	0.67 ± 0.82	0.89 ± 0.60	1.0 ± 0.71	0.582
ASES	88.73 ± 14.69	70.51 ± 17.1	65.52 ± 20.03	0.064
UCLA	29.2 ± 3.11	25.44 ± 6.64	23.2 ± 8.29	0.177
SST	10.2 ± 1.79	7.0 ± 2.87	6.0 ± 3.54	0.025

*n.s* no significant, *VAS* visual analogue scale, *ASES* American Shoulder and Elbow Surgeons Assessment Form, *UCLA:* University of California at Los Angeles Shoulder Rating Scale, *SST* Simple Shoulder Test score

In the histology of the early (prodromal) stage, chronic inflammation within fatty marrow and healing fracture callus was found. No evidence of AVN, infection or neuropathy was observed In the advanced stage, Intraoperatively, progressive destructive and sclerotic humeral head covered with fibrotic synovium was observed in the advanced stage. Pathologic finding showed chronic inflammation composed of many macrophages, several foreign body type multinuclear giant cells and fibrosis (H&E stain, X100). Also, soft tissue adjacent bone showed chondroid metaplasia and chronic inflammation (H&E stain, X400) (Fig. 6).

## Discussion

The most important finding of our study was that shoulder arthroplasty provides satisfactory mid-term clinical outcomes in the RPDA of shoulder. Without any definite underlying pathogenesis, both TSA and rTSA significantly improved functional and radiological outcomes in these RPDA patients. Early diagnosis is essential as it may help reduce the complexity and extent of surgical management. Delayed diagnosis and further deterioration of the humeral head and glenoid made its treatment more difficult. Early detection and prompt treatment of this RPDA showed favorable outcomes without severe complications.

RPDA is a rare condition characterized by rapid deterioration of the shoulder joint. This rare disease entity has been more frequently observed in elderly women, mainly due to the aging population or a history of trauma. Clinical features of RPDA are distinct from those of other types of shoulder arthritis. Patients typically present with severe shoulder pain and rapid functional deterioration, often progressing to pseudoparalysis within months. Unlike conventional osteoarthritis or cuff tear arthropathy, which progress gradually over years, RPDA demonstrates a rapid clinical course that is disproportionate to the relatively subtle radiographic findings at the early stage. Moreover, inflammatory markers remain normal, which differentiates it from rheumatoid arthritis or septic arthritis. Histologic findings also show reactive synovitis and fibrosis rather than specific inflammatory or infectious changes. However, diagnosing RPDA can be challenging as it may be confused with other arthropathies, infections, or osteonecrosis. This condition often leads to severe pain and significant loss of function over a short period of time. The typical findings was rapid flattening and collapse of the humerus head within a short time periods after the onset of symptoms. However, symptom duration after the initial symptom’s onset, was significantly variable in our study. In one patient, the interval from symptom onset to radiographically confirmed humeral head collapse was 60 months, which may appear inconsistent with the definition of rapid progression; however, this case demonstrated a prolonged prodromal phase with slowly evolving symptoms, followed by an accelerated phase of radiographic deterioration culminating in humeral head collapse, and should therefore be considered an atypical presentation of RPDA. Another notable finding in our series was that all patients developed RPDA in the dominant arm, highlighting the clinical significance of this condition in functional outcomes. In one patient, RPDA developed in the contralateral shoulder after undergoing resection arthroplasty, ultimately requiring RTSA. These findings indicate the necessity of careful follow-up, particularly in patients with unilateral disease affecting the non-dominant side, as progression to the dominant arm may critically impair daily function. Surgical intervention, such as shoulder arthroplasty, should be considered, especially when conservative treatment fails. However, it can be challenging to diagnose early due to the lack of specific early radiographic changes. Only, MRI can detect the trace of the RPDA in the early stage.

RPDA has been commonly seen in hip joint and Postel and Kerboull [4] were first to described this condition in 1970. It is difficult to differentiate RPDA radiographically from rheumatoid disease, pyogenic arthritis, drug-induced arthropathy, cuff-tear arthropathy, osteoarthrosis, avascular osteonecrosis. RPDA involves often elderly female with rapid destruction of bone without any significant inflammatory changes. However, many of our patients had history of trauma or arthroscopic rotator cuff surgery. Kekatpure et al. [12] reported 9 cases of RPDA, out of which 7 patients had rotator cuff tears and 2 patients had supraspinatus tendinosis. Only 3 out of 7 patients had retracted tears upto glenoid margin suggesting the etiology of RPDA to be different from cuff tear arthropathy. The primary cause of humeral head osteonecrosis following rotator cuff repair is most likely attributable to vascular disruption. Yamamoto et al. [13, 14] reported that subchondral insufficiency due to osteopenia might be responsible for RPDA of hip. Tokuya et al. [15] presented 1 case of RPDA with subchondral humeral head fracture with glenoid bone insufficiency and suggested that RPDA resulted from a subchondral humeral head fracture. Although the exact mechanism remains uncertain. One possible cause of RPDA is thought to be subchondral fracture. RPDA following rotator cuff repair is a rare but severe complication. In our series, nearly half of the patients did have any rotator cuff surgery or some trauma history. It can occur due to disruption of the intraosseous vascular network during surgical procedures, particularly with suture anchor placement, which can damage the blood supply to the humeral head [16]. Although rapid progressive osteonecrosis of the humeral head was observed after arthroscopic rotator cuff repair, the study found no definitive causal relationship between the procedure and the development of osteonecrosis. However, the authors emphasized that older patients or those with compromised bone quality may be at higher risk, and careful postoperative monitoring should be considered necessary [17].

Histologic findings typically reveal varying degrees of synovitis with mild acute and chronic features, often described as reactive rather than inflammatory, along with extensive capsular fibrosis and occasional subchondral fractures. Areas of bone necrosis may be present, ranging from minimal to extensive. Overall, the histologic appearance resembles that of conventional osteoarthritis [13, 18]. Osteoporosis-induced subchondral insufficiency fracture may play a significant role in the pathogenesis of these disorder. Mature, activated osteoclasts have been found in the synovium and bone surfaces of the hip. According to study of Kim et al. [7], T1-weighted MRI showed a subchondral fracture (100%) of low signal intensity with associated bone marrow edema. Histologically, chronic inflammation of the synovium and osteocytes in the lacunae, as well as callus formation, were observed along the subchondral fracture. It is similar radiographically, in its final stages, to rotator cuff tear arthropathy and Milwaukee shoulder. Sometimes, RPDA shares certain gross, microscopic and synovial fluid features of those disorders. Crystals or large cuff defects, as well as the typical findings of osteonecrosis and osteoarthritis, may be notably absent. Milwaukee shoulder syndrome may also represent a subtype of RPDA.

The radiologic manifestations of RPDA in both the hip and shoulder are characterized by rapid chondrolysis, followed by accelerated and extensive subchondral bone resorption or destruction. Radiographic progression is typically classified into two distinct stages: (a)

an early chondrolysis stage, and (b) a late subchondral destruction stage [18]. In our study, MRI showed a bone marrow edema with an associated subchondral low-intensity band on the T1-weighted image at the early period of RPDA. Through the MRI evaluation, we could find the early stage of RPDA. After we added the early symptomatic stage with subchondral edema on the MRI, we suggest 3 radiologic stages: (1) an early prodromal stage, (2) an mid chondrolysis stage, and (3) a late subchondral destruction stage (Table 4).

**Table 4:** Radiologic classification of rapidly progressive destructive arthropathy

Stage	Radiologic Features	Rotator Cuff Status	Preferred Treatment
Stage 1 (Early Prodromal)	Focal bone marrow edema, small subchondral fracture (MRI/X-ray)	Intact or minimally damaged cuff	TSA or RTSA depending on tendon quality
Stage 2 (Mid Chondrolysis)	Collapse < 50% of humeral head; whole bone marrow edema	Partial or full-thickness tear with poor quality	RTSA
Stage 3 (Late Subchondral Destruction)	Collapse > 50%, metaphyseal bone loss, proximal humerus involvement	Absent rotator cuff	Conventional RTSA with long stem

Tokuya et al. [15] presented 1 case of RPDA with subchondral humeral head fracture with glenoid bone insufficiency and suggested that RPDA resulted from a subchondral humeral head fracture. McCarty et al. [9] coined the term “the Milwaukee shoulder syndrome”, which is characterized by involvement of aged female around 63 to 90 years with pain in shoulder, reduced mobility, complete rotator cuff tear, abundant joint effusion containing hydroxyapatite crystals and a high level of activated collagenase. We did not observe any such crystals in histological analysis of intraoperative specimen. Also, arthroscopic rotator cuff surgery has been reported to develop these rapid progressive osteonecrosis of the humeral head rarely. Kim et al. [17] reported that rapid progressive osteonecrosis of the humeral head after arthroscopic rotator cuff surgery was developed within 12 months after index surgery at the dominant side of elderly women.

In our series, advanced humeral head collapse associated with RPDA was occasionally observed, and in rare cases, it was accompanied by subchondral involvement of the glenoid bone. In three patients, secondary glenoid bone defects precluded the use of conventional TSA. A distinctive pattern of humeral head collapse, referred to as a “chopped humeral head” as previously described by Kekatpure et al. [12] was identified. The mean age of these patients was over 70 years, and they commonly presented with massive rotator cuff tears or severely degenerated rotator cuff tendons—factors that are considered relative contraindications for conventional TSA. Consequently, RTSA was performed in all cases except for two patients who underwent TSA in an effort to minimize potential complications.

Another consideration of the arthroplasty in late stage of the RPDA was the stem length. In these RPDA, MRI showed bone marrow changes and bone loss in the proximal humerus. Short stem humeral implants provided good clinical outcomes with low revision rates [19, 20]. However, short stem potentially carry the risk of malalignment and no rigid fixation in the late advanced stages of RPDA. In the late advanced stages, destructive changes were often extended to the proximal metaphysis and metaphyseal stability of the short stem could compromise fixation. Also, the humeral cortex often becomes highly sclerotic, which may increase the risk of intraoperative cortical fracture during stem insertion. Surgeons should therefore exercise great caution during stem preparation in this stage to avoid iatrogenic complications. In the early and mid-stages in which the proximal metaphysis was not damaged, the short stem could provide the excellent clinical outcomes. However, long term follow-up study will be necessary to evaluate the clinical and radiologic results in RPDA. To the best of our knowledge, this is the first case series to classify and analyze RPDA based on disease stage. Good clinical outcomes of arthroplasty of hip are reported for RPDA, but there is a little case series for shoulder and hence we could not compare our outcomes. However, all the patients in our series had significantly improved clinical outcomes after shoulder arthroplasty for RPDA at 2 year follow-up suggesting promising results for this condition. These findings should be interpreted with caution due to the small sample size and the limited number of TSA cases.

Limitations of this study were as follows. First, this study was originally retrospective study. Second, the number of each patient group was too small, so there was a limit to calculating and comparing the clinical outcomes by dividing into groups. However, in the early phase, we could not suspect the rapid progression of this disease entity. In these patients, early diagnosis may allow for discussions regarding the potential for rapid disease progression, and timely intervention could help prevent further deterioration of the shoulder joint. Third, the follow-up period in this study was relatively short, limited to two years. Long-term follow-up data regarding potential complications and stem stability would provide valuable insights into optimizing treatment strategies for patients with RPDA.

## Conclusion

RPDA of the humeral head is a unique condition that should be distinguished from early-stage disease, which is often misdiagnosed. Although there may be no specific early radiologic changes in the humeral head, careful radiologic follow-up is essential. For patients with RPDA of the shoulder, the primary treatment is joint replacement (either TSA or RTSA) depending on the condition of the rotator cuff and secondary involvement of the glenoid. Ideally, shoulder arthroplasty should be performed before the onset of severe glenoid erosion or humeral head collapse, which are common in the later stages of RPDA. Close monitoring of high-risk patients is crucial to enable timely surgical intervention and to reduce operative time and the complexity of reconstruction.

## Data Availability

The datasets generated and/or analysed during the current study are not publicly available due to institutional policy and patient privacy protection, but are available from the corresponding author on reasonable request.
